# Thermal Energy Storage and Heat Transfer of Nano-Enhanced Phase Change Material (NePCM) in a Shell and Tube Thermal Energy Storage (TES) Unit with a Partial Layer of Eccentric Copper Foam

**DOI:** 10.3390/molecules26051491

**Published:** 2021-03-09

**Authors:** Mohammad Ghalambaz, Seyed Abdollah Mansouri Mehryan, Kasra Ayoubi Ayoubloo, Ahmad Hajjar, Mohamad El Kadri, Obai Younis, Mohsen Saffari Pour, Christopher Hulme-Smith

**Affiliations:** 1 Metamaterials for Mechanical, Biomechanical and Multiphysical Applications Research Group, Ton Duc Thang University, Ho Chi Minh City 758307, Vietnam; mohammad.ghalambaz@tdtu.edu.vn; 2 Department for Management of Science and Technology Development, Faculty of Applied Sciences, Ton Duc Thang University, Ho Chi Minh City 758307, Vietnam; 3 Young Researchers and Elite Club, Yasooj Branch, Islamic Azad University, Yasooj 7591493686, Iran; alal171366244@gmail.com; 4 Department of Mechanical Engineering, Shahid Chamran University of Ahvaz, Ahvaz 61355, Iran; kasra.ayoubi@yahoo.com; 5 ECAM Lyon, LabECAM, Université de Lyon, 69005 Lyon, France; ahmad.hajjar@ecam.fr; 6 Centre Scientifique et Technique du Bâtiment, 44323 Nantes, France; mohamad.elkadri@hotmail.com; 7 Laboratoire de Génie des Procédés Chimiques, Université Ferhat Abbas Sétif-1, Sétif 19000, Algeria; 8 Department of Mechanical Engineering, College of Engineering at Wadi Addwaser, Prince Sattam Bin Abdulaziz University, Wadi Addwaser 11991, Saudi Arabia; oubeytaha@hotmail.com; 9 Department of Mechanical Engineering, Faculty of Engineering, University of Khartoum, Khartoum 11111, Sudan; 10 Department of Mechanical Engineering, Faculty of Engineering, Shahid Bahonar University of Kerman, Kerman 7616913439, Iran; 11 Department of Materials Science and Engineering, KTH Royal Institute of Technology, SE-100 44 Stockholm, Sweden

**Keywords:** latent heat thermal energy storage, annuli enclosure, graphene oxide nanoparticles, copper metal foam, thermal enhancement

## Abstract

Thermal energy storage units conventionally have the drawback of slow charging response. Thus, heat transfer enhancement techniques are required to reduce charging time. Using nanoadditives is a promising approach to enhance the heat transfer and energy storage response time of materials that store heat by undergoing a reversible phase change, so-called phase change materials. In the present study, a combination of such materials enhanced with the addition of nanometer-scale graphene oxide particles (called nano-enhanced phase change materials) and a layer of a copper foam is proposed to improve the thermal performance of a shell-and-tube latent heat thermal energy storage (LHTES) unit filled with capric acid. Both graphene oxide and copper nanoparticles were tested as the nanometer-scale additives. A geometrically nonuniform layer of copper foam was placed over the hot tube inside the unit. The metal foam layer can improve heat transfer with an increase of the composite thermal conductivity. However, it suppressed the natural convection flows and could reduce heat transfer in the molten regions. Thus, a metal foam layer with a nonuniform shape can maximize thermal conductivity in conduction-dominant regions and minimize its adverse impacts on natural convection flows. The heat transfer was modeled using partial differential equations for conservations of momentum and heat. The finite element method was used to solve the partial differential equations. A backward differential formula was used to control the accuracy and convergence of the solution automatically. Mesh adaptation was applied to increase the mesh resolution at the interface between phases and improve the quality and stability of the solution. The impact of the eccentricity and porosity of the metal foam layer and the volume fraction of nanoparticles on the energy storage and the thermal performance of the LHTES unit was addressed. The layer of the metal foam notably improves the response time of the LHTES unit, and a 10% eccentricity of the porous layer toward the bottom improved the response time of the LHTES unit by 50%. The presence of nanoadditives could reduce the response time (melting time) of the LHTES unit by 12%, and copper nanoparticles were slightly better than graphene oxide particles in terms of heat transfer enhancement. The design parameters of the eccentricity, porosity, and volume fraction of nanoparticles had minimal impact on the thermal energy storage capacity of the LHTES unit, while their impact on the melting time (response time) was significant. Thus, a combination of the enhancement method could practically reduce the thermal charging time of an LHTES unit without a significant increase in its size.

## 1. Introduction

Eighty-one percent of energy consumption was produced from fossil fuel resources in 2017 [1]. Energy lost as waste heat accounts for 12.2% of global energy consumption [2,3]. Renewable energy is under rapid development, so it can replace fossil fuel energy and reduce global warming gas emissions. However, different types of renewable energy are generally intermittent and fluctuant, so it is impossible to use them continuously. Furthermore, there is a discrepancy between the supply and the demand which must be reconciled. One potential solution is to store surplus energy as heat and release it when there is a deficit in power generation. This is known as thermal energy storage (TES). One way to achieve this is to use excess energy to force a material to undergo an endothermic phase change, which can be reversed to release latent heat when needed. This is the concept of latent heat thermal energy storage (LHTES).

One of the advantages of LHTES is its large capacity to store energy, compared with alternative concepts such as sensible heat TES. However, the concept suffers from a low thermal response, which is the main disadvantage in commercialization and large-scale applications of LHTES. The heat transfer efficiency is limited, and the charge/discharge process is lengthened in most materials because of poor thermal conductivity.

To enhance the thermal response of LHTES, various techniques have been proposed, such as the use of a finned unit to increase total surface areas [4,5], direct contact heat transfer [6], and the modification of materials with a porous matrix [7,8]. Several studies have demonstrated that the time required to store and discharge heat is reduced by using foams, such as a copper foam bonded to a material that undergoes the phase change [9], a paraffin/copper foam and paraffin/nickel foam composite [10], and conductive foams and finned pipes [11].

During charging, the primary heat transfer mechanisms are conduction and (natural) convection [12]. Therefore, the performance of any system is dependent on the geometry. For example, in a shell-and-tube heat exchanger, in which a central tube extracts a head from a molten material held in a container (“shell”), more heat is transferred when the axis of both the shell and the tube is horizontal, but the maximum rate of heat transfer is highest when both were vertical [13]. Another study showed that if enough molten material is present in a horizontal shell with a finned copper tube, convection dominates heat transfer [14].

A very important parameter in the design of shell-and-tube TES systems is the eccentricity of the tube [15]. In a system that uses a horizontal shell of a storage material, a reduction in eccentric distance leads to an improvement in the melting rate and an increase in the rate of heat transfer by natural convection [16]. The orientation of the tube can be changed independently of the shell: the orientation of the tube in a horizontal shell system impacts the rate at which heat can be stored (the charging speed) [17].

Using an extended surface or fins can also improve the thermal performance of LHTES systems by providing a higher surface area across which heat may be transferred. Numerous researchers have studied potential configurations of finned heat exchangers such as tree-shaped fins [18], pin fin heat sinks [19,20], and plate-finned heat exchangers [21]. A plate-fin unit with a length of 2 mm for rapid heat storage/release using paraffin was studied numerically. When temperature differences in the system are less than 20 °C, the fins are vital to the energy storage performance [22]. In a fin-and-tube heat exchanger, the use of fins decreases the time for both melting and the solidification of the storage material, independent of the flow regime and pitch of the fins [23]. In a plate-fin-type system, it has been found that the Stefan number, the Rayleigh number, the Nusselt number, and the Fourier number are important factors [24].

Heat sinks with pin fins and materials that undergo phase changes have been utilized widely in the cooling of electronic products. The base temperature and the heat exchanger operating time can be decreased significantly by raising the number, the height, and the thickness of the fins [25,26].

Metal foams could increase the thermal conductivity in LHTES and thereby enhance heat transfer. In one study using expanded graphite and metal foams, an interconnected structure of metal foams enhances heat transfer but reduces convection in the molten material [27]. Increasing the porosity linearly from the bottom of the system to the top can shorten the time required to melt the storage material and improve the heat transfer performance, compared to the case of a constant porosity of the investigated melting in a phase change material (PCM) saturated in an irregular geometry. Mesalhy et al. [28] investigated an irregular geometry filled with a high thermal-conductive porous matrix. The authors showed an increase in the melting rate when the porosity of the matrix decreases, but the convection is hindered. Overall, it is concluded from the literature that the use of a solid matrix with high thermal conductivity and high porosity is the best technique to improve storage response in LHTES.

The net useful energy during charging and discharging periods has been estimated by performing energy and exergy analyses of shell- and tube-type LHTES units for a medium temperature solar thermal power plant (~200 °C) [29]. Similarly, the behavior of a LHTES system in the presence and in the absence of an aluminum foam has been studied. It was shown that the presence of foam accelerates the phase change and the heat transfer of the LHTES system and that the melting time is shown to increase when the porosity of the foam increases [30].

Adding nanoparticles to materials that undergo a phase change in a porous energy storage system decreases the solidification time by up to 23.5%. However, the solidification time decreases by 14% when adding nanoparticles with volume fractions up to 4% [31]. For example, by adding boron nitride nanoparticles to calcium chloride hexahydrate, a thermal conductivity can be 71.9% higher than that with calcium chloride hexahydrat alone. However, the specific heat capacity and the latent heat of fusion decrease 60.9% and 11.1%, respectively, when the nanoparticles are added. Incorporating alumina nanoparticles to a material inside a triplex tube has little effect at the beginning of the solidification process. However, the rate of solidification increases with the volume fraction of nanoparticles [32].

The use of a metal foam, fins, and nanoparticles were each studied in the thermal management of a lithium-ion battery. The foam gave a bigger performance boost than either the fins or the nanoparticles [33]. Separately, a multiple-segment metal foam with an average porosity of 0.95 and an incorporation of 5 vol.% nanoparticles in a tube-and-shell LHTES system reduces solidification time by up to 94%, compared to the case of a single pure storage material. The effect is dependent on the number of cascaded foam segments and the number of different materials that are able to undergo a phase change [34]. In a separate study, the simultaneous use of a porous foam and nanoparticles reduces solidification time by 21.4%, compared to the use of a pure material [35].

Metal foams can improve the response time of LHTES units. Using nanoparticles can also improve thermal conductivity and heat transfer. However, a porous matrix limits natural convection flows. A dispersion of nanoparticles raises the dynamic viscosity [36] and, consequently, reduces natural convection flows. Moreover, the addition of metal foams and nanoadditives will increase the masses of LHTES units. In addition, since they do not contribute to latent heat energy storage, the overall heat capacity of the LHTES units can be reduced. The proper design of an LHTES system is a crucial task to benefit from the heat transfer improvement of metal foams and nanoadditives but to avoid overweighting the system and reducing thermal storage capacity to unacceptably low levels.

The present study addresses the impact of employing a layer of a porous medium over the inner hot pipe of an LHTES unit in the presence of nanoadditives. The porous layer can be placed with eccentricity toward the bottom to keep the advantage of natural convection flows at the top region of the TES unit while enhancing the conduction heat transfer at the bottom solid regions. The degree to which a metal foam layer affects the response (melting) time and the effect of nanoparticle additions on the heat transfer rate are investigated. The effectiveness of nanoparticles in the presence of the convective flows and the metal foam and the melting behavior of such a system will also be considered. A mathematical model will be developed to consider these effects.

## 2. Physical Model and Mathematical Formulation

### 2.1. Model Description

**Figure 1:**
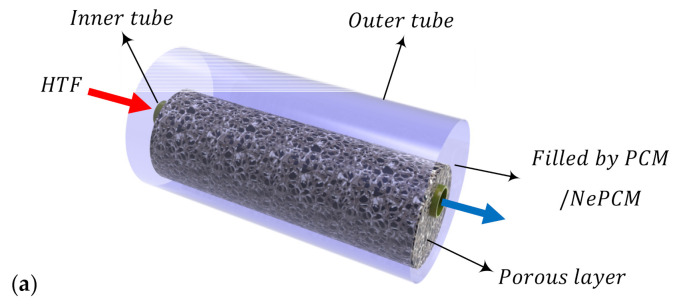

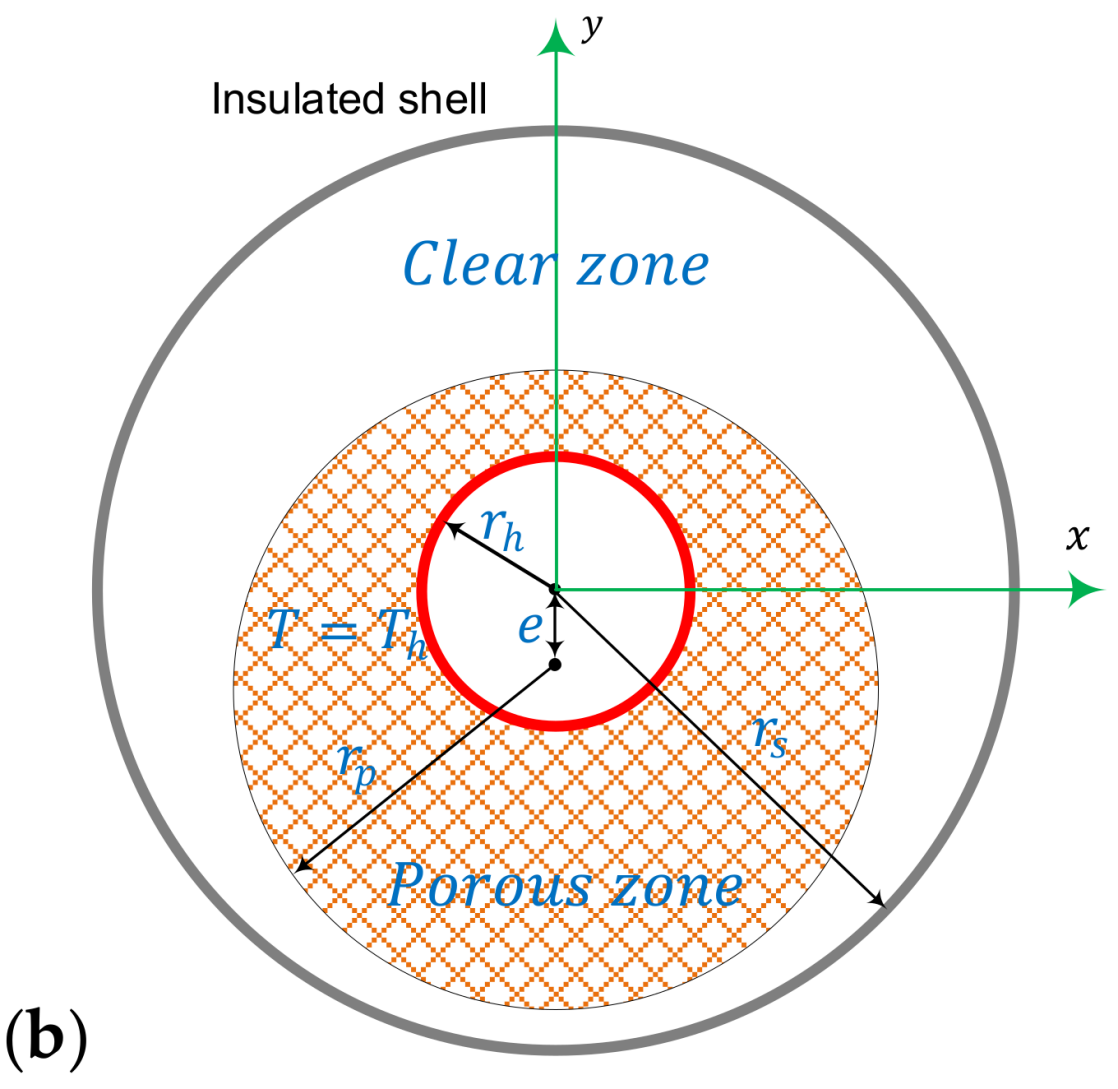
(**a**) Schematic representation of the three-dimensional thermal storage unit; and (**b**) the cross-section of the energy storage unit (and the computational domain).

Figure 1a illustrates the structure of a TES unit utilized in this work. The inner pipe in the system is surrounded by a porous annulus with a permeability, *κ*, porosity, *ε*, and eccentricity, *e*. The velocity of a heat transfer fluid flowing in the inner pipe is high. In addition, the inner pipe employed in the system has a low thickness and a high thermal conductivity. Hence, it is reasonable to assume that the tube temperature is fixed at *T*_h_. The pores of the porous annulus and the void space between the porous annulus and the outer shell of the system are occupied with a nano-enhanced phase change material (NePCM) with homogenously dispersed nanoadditives of cooper or graphene oxide. The volume fraction of the nanoparticles in the base phase change material is *σ*. The outer shell of the system is well insulated, and the NePCM is initially at a supercooled temperature *T*_c_< *T*_h_. The geometrical characteristics of the cross-section of the unit (Figure 1b) are *r*_s_ = 30 mm, *r*_h_ = 2*r*_s_/5, and *r*_p_ (*e* = 0) = 3*r*_s_/4. Table 1 lists the thermal properties of the PCM, the nanoadditives, and the metal foam.

### 2.2. Governing Equations

Liquid flow in the clear and porous domains can be solved by the controlling equations expressed below [39,40,41]:

Continuity equation:

(1) $$\frac{\partial u}{\partial x}+\frac{\partial v}{\partial y}=0$$

Momentum equation in the x direction:

(2) $$\frac{\rho_{\mathrm{NeP},l}}{\varepsilon_{j}}\frac{\partial u}{\partial t}+\frac{\rho_{\mathrm{NeP},l}}{\varepsilon_{j}^{2}}\left(u\frac{\partial u}{\partial x}+v\frac{\partial u}{\partial y}\right)=-\frac{\partial p}{\partial x}+\frac{\mu_{\mathrm{NeP},l}}{\varepsilon_{j}}\left(\frac{\partial^{2}u}{\partial x^{2}}+\frac{\partial^{2}u}{\partial y^{2}}\right)-\left(\frac{\mu_{\mathrm{NeP},l}}{\kappa_{j}}+s\left(T\right)\right)u$$

Momentum equation in the y direction:

(3) $$\frac{\rho_{\mathrm{NeP},l}}{\varepsilon_{j}}\frac{\partial v}{\partial t}+\frac{\rho_{\mathrm{NeP},l}}{\varepsilon_{j}^{2}}\left(u\frac{\partial v}{\partial x}+v\frac{\partial v}{\partial y}\right)=-\frac{\partial p}{\partial y}+\frac{\mu_{\mathrm{NeP},l}}{\varepsilon_{j}}\left(\frac{\partial^{2}v}{\partial x^{2}}+\frac{\partial^{2}v}{\partial y^{2}}\right)-\left(\frac{\mu_{\mathrm{NeP},l}}{\kappa_{j}}+s\left(T\right)\right)v+\rho_{\mathrm{NeP},l}\beta_{\mathrm{NeP},l}g\left(T-T_{\mathrm{fu}}\right)$$

where *u* and *v* are the velocity components for an NePCM, and *t* is the time; The symbols *ρ* and *μ* are the density and the dynamic viscosity, respectively; κ and ε are the permeability and porosity of the metal foam, respectively; *s*(*T*) is a source term which forces the velocities to zero in the solid domain; the subscripts of NeP and l indicate the NePCM and liquid state for the NePCM, respectively. In the above equations, spatial functions for the porosity and permeability *ε_j_* and *κ_j_* were defined as:

(4) $$\varepsilon_{j}=\left\{\begin{array}{l} \varepsilon \;j=1\\ 1\;j=2 \end{array}\right.,\kappa_{j}=\left\{\begin{array}{l} \kappa \;j=1\\ \infty \;j=2 \end{array}\right.$$

where 1 and 2 in the above functions denote the porous and clear zones, respectively; *κ* is the permeability of the porous zone for a porous medium with a pore diameter, *d*_p_, and ligament diameter *d*_l_, was given by [42,43]:

(5) $$\kappa =d_{p}^{2}\frac{73\times 10^{-5}}{{\left(1-\varepsilon \right)}^{0.224}}\left[d_{l}^{-1.11}d_{p}^{1.11}\right]$$

(6) $$\left[d_{l}^{-1.11}d_{p}^{1.11}\right]=1.18{\left(\frac{1-\varepsilon }{3\pi }\right)}^{0.5}{\left[1-\exp\left(-\left(1-\varepsilon \right)/0.04\right)\right]}^{-1}$$

where

(7) $$d_{p}=254\times 10^{-4}\omega^{-1}\left(\mathrm{PPI}\right)$$

Moreover, the momentum sink term, i.e., *s*(*T*), was defined as the following:

(8) $$s\left(T\right)=A_{\mathrm{mush}}\frac{{\left(1-\eta \left(T\right)\right)}^{2}}{\eta {\left(T\right)}^{3}+\vartheta }$$

where *A*_mush_ is a large number O(10^6^) and *ϑ* is a small number O(10^−3^); $\eta \left(T\right)$ is written as:

(9) $$\eta \left(T\right)=\left\{\begin{array}{ll} 0 & T<T_{\mathrm{fu}}-\delta T/2\\ \frac{T-T_{\mathrm{fu}}}{\delta T}+\frac{1}{2} & T_{\mathrm{fu}}-\delta T/2<T<T_{\mathrm{fu}}+\delta T/2\\ 1 & T>T_{\mathrm{fu}}+\delta T/2 \end{array}\right.$$

(10) $${\left(\rho C_{p}\right)}_{m}\frac{\partial T}{\partial t}+{\left(\rho C_{p}\right)}_{\mathrm{NeP},l}\left(u\frac{\partial T}{\partial x}+v\frac{\partial T}{\partial y}\right)=\lambda_{m,j}\left(\frac{\partial^{2}T}{\partial x^{2}}+\frac{\partial^{2}T}{\partial y^{2}}\right)-\rho_{\mathrm{NeP},l}h_{\mathrm{sf},\mathrm{NeP}}\varepsilon_{j}\frac{\partial \eta \left(T\right)}{\partial t}$$

where

(11) $${\left(\rho C_{p}\right)}_{m}=\eta {\left(\rho C_{p}\right)}_{m,\mathrm{Nep},l}+\left(1-\eta \right){\left(\rho C_{p}\right)}_{m,\mathrm{NeP},s}$$

where

(12) $${\left(\rho C_{p}\right)}_{m,\mathrm{NeP},i}=(1-\varepsilon_{j}){\left(\rho C_{p}\right)}_{m}+\varepsilon_{j}{\left(\rho C_{p}\right)}_{\mathrm{NeP},i}$$

where *i* describes the different states of the NePCM, *C*_p_ is the heat capacity, and *η* is the liquid volume fraction; the subscripts of m and s denote the porous matrix and solid state of the NePCM, respectively. The thermal conductivity of the zones, *λ*_m,*j*_, was described as:

(13) $$\lambda_{m,j}=\left\{\begin{array}{l} \lambda_{m,\mathrm{eff}}\;j=1\\ \lambda_{\mathrm{NeP}}\;j=2 \end{array}\right.$$

where

(14) $$\lambda_{m,\mathrm{eff}}=\eta \lambda_{m,\mathrm{eff},l}+\left(1-\eta \right)\lambda_{m,\mathrm{eff},s}$$

(15) $$\lambda_{\mathrm{NeP}}=\eta \lambda_{\mathrm{NeP},l}+\left(1-\eta \right)\lambda_{\mathrm{NeP},s}$$

A large number of relationships which can be used to evaluate the effective thermal conductivity of the porous medium are presented in the literature [44]. In this study, the following relation, which takes into account the major characteristics of the porous matrix, was employed as following [28,45]:

(16) $$\lambda_{m,\mathrm{eff},i}=\frac{\left[\lambda_{\mathrm{NeP},i}+\pi \left(\sqrt{\chi }-\chi \right)\Delta \lambda \right]\left[\lambda_{\mathrm{NeP},i}+\left(\chi \pi \right)\Delta \lambda \right]}{\lambda_{\mathrm{NeP},i}+\left[\frac{4}{3}\sqrt{\chi }\left(1-\varepsilon \right)+\pi \sqrt{\chi }-\left(1-\varepsilon \right)\right]\Delta \lambda }$$

where

(17) $$\chi =\frac{1-\varepsilon }{3\pi },\;\Delta \lambda =\lambda_{m}-\lambda_{\mathrm{NeP},i}$$

### 2.3. The Thermal and Physical Specifications of the PCM Enhanced with Nanoparticles

The density of the PCM enhanced with nanoparticles, *ρ*_NeP_, was expressed as:

(18) $$\rho_{\mathrm{NeP}}=\left(1-\sigma \right)\rho_{\mathrm{PCM}}+\sigma \rho_{\mathrm{na}}$$

(19) $$\rho_{\mathrm{PCM}}\left(T\right)=\rho_{\mathrm{PCM},l}\eta \left(T\right)+\left(1-\eta \left(T\right)\right)\rho_{\mathrm{PCM},s}$$

where *σ* is the concentration of the nanoadditives. The dynamic viscosity of the PCM enhanced with nanoparticles, *µ*_NeP,1_, was written as [36]:

(20) $$\mu_{\mathrm{NeP},l}=\mu_{\mathrm{PCM},l}{\left(1-\sigma \right)}^{-2.5}$$

The thermal-volume expansion coefficient of the PCM enhanced with nanoparticles, *β*_NeP,1_, is expressed as:

(21) $$\rho_{\mathrm{NeP},l}\beta_{\mathrm{NeP},l}=\left(1-\sigma \right)\rho_{\mathrm{PCM},l}\beta_{\mathrm{PCM},l}+\sigma \rho_{\mathrm{na}}\beta_{\mathrm{na}}$$

The effective thermal conductivity of the enhanced PCM, *λ*_NeP,1_, was expressed as:

(22) $$\frac{\lambda_{\mathrm{NeP},i}}{\lambda_{\mathrm{PCM},i}}=\frac{\left(\lambda_{\mathrm{na}}+2\lambda_{\mathrm{PCM},i}\right)-2\sigma \left(\lambda_{\mathrm{PCM},i}-\lambda_{\mathrm{na}}\right)}{\left(\lambda_{\mathrm{na}}+2\lambda_{\mathrm{PCM},i}\right)+\sigma \left(\lambda_{\mathrm{PCM},i}-\lambda_{\mathrm{na}}\right)}$$

The heat capacity of the PCM enhanced with nanoparticles, *C*_p,NeP_, was described as:

(23) $$\rho_{\mathrm{NeP}}C_{p,}{}_{\mathrm{NeP}}=\left(1-\sigma \right)\rho_{\mathrm{PCM}}C_{p,}{}_{\mathrm{PCM}}+\sigma \rho_{\mathrm{na}}C_{p}{}_{,\mathrm{na}}$$

(24) $$\rho_{\mathrm{PCM}}C_{p,}{}_{\mathrm{PCM}}\left(T\right)=\rho_{\mathrm{PCM},l}C_{p}{}_{,\mathrm{PCM},l}\eta \left(T\right)+\left(1-\eta \left(T\right)\right)\rho_{\mathrm{PCM},s}C_{p}{}_{,\mathrm{PCM},s}$$

the heat latent of the composite material, *h*_sf,NeP_, was written as:

(25) $$\rho_{\mathrm{NeP},l}h_{\mathrm{sf},\mathrm{NeP}}=\left(1-\sigma \right)\rho_{\mathrm{PCM},l}h_{\mathrm{sf},\mathrm{PCM}}$$

### 2.4. Boundary Conditions

The controlling boundary conditions are as following:

(a) at the interface of the porous and clear zones:
  (26) $${\left.T\right|}_{\;\mathrm{cz}}={\left.T\right|}_{\mathrm{pz}},\;{\left.k_{\mathrm{PCM}}\frac{\partial T}{\partial n}\right|}_{\;\mathrm{cz}}={\left.k_{m,\mathrm{eff}}\frac{\partial T}{\partial n}\right|}_{\mathrm{pz}}$$
(b) at the inner tube and shell:
  (27) $$T=T_{h},\;u=0,\;\;v=0$$
  (28) $$\frac{\partial T}{\partial n}=0,\;u=0,\;\;v=0\;$$
  where *T*_h_ = 316 K. The initial temperature was also considered as 304 K which is one degree below the fusion temperature.

### 2.5. Characteristic Parameters

The energy stored in the system with the nanoparticle-enhanced PCM, *ES*, was defined by:

(29) $$ES=\int_{A}{\left(\rho C_{p}\right)}_{m,\mathrm{NeP}}\left(T-T_{c}\right)\;dA+\int_{A}\rho_{\mathrm{NeP},l}h_{\mathrm{sf},\mathrm{NeP}}\varepsilon_{j}dA$$

Finally, the melting volume fraction, *MVF*, was evaluated as:

(30) $$MVF=\frac{\int_{A}^{}\varepsilon_{j}\eta dA}{\int_{A}^{}\varepsilon_{j}dA}$$

## 3. Numerical Method

### 3.1. Solution Approach

The system is divided into three regions: the first region where the PCM is completely solid, the second region where it is completely molten, and the third region where solid and liquid states of the material coexist (the mushy zone). In order to distinguish these three zones, the fusion temperature and the melting temperature window, *δT,* are used. Melting and solidification can be described by a movement of the interfaces between these three zones and a change in the phase fraction of liquid within the mushy zone. The melting process can be modeled by using source terms for momentum, *s*(*T*), and energy, via the rate of latent heat generation and is proportional to the rate of change of fraction of liquid, *∂η(T)*/*∂t*, which are described in the previous section. In addition, the velocity components are controlled by the source term, *s*(*T*). This source term acts as a virtual porous material with variable permeability, as introduced in Equation (5). The magnitude of *s*(*T*) approaches zero in the liquid domain (no force on the fluid) and has a very large number (impermeable medium) in the solid domain. Since the velocity of liquid reaches zero in the solid domain through the mushy zone, a high-velocity gradient is seen inside the mushy zone. The energy source term, *∂η(T)*/*∂t*, acts as a heat sink within the mushy zone, controls the latent heat abortion and results in a high-temperature gradient. To calculate the high gradients in the mushy zone, a very fine mesh is vital. However, using such a fine mesh throughout the domain would be prohibitive in terms of computational expense. Therefore, a mesh adaptation technique was used.

A Galerkin finite element method technique was used to solve the nonlinear differential equations in this study. The phase change equations were implemented by user defined codes. According to this method, the equations and the corresponding boundary and initial conditions were transformed to a weak form and solved by the Galerkin finite element method. The residual equations based on shape functions were introduced and solved iteratively. The numerical approach could be found in [46,47], and it was not repeated here for the sake of brevity.

A dummy phase field variable, *η*_0_, was introduced that acted as a criterion for grid adaption, i.e., only when *η*_0_ = 1, the mesh adaptation will occur. *η*_0_ was defined as:

(31) $$\eta_{0}\left(T\right)=\left\{\begin{array}{l} 0\;T\leq T_{fu}-3\delta T/2\\ 1\;T_{fu}-3\delta T/2<T<T_{fu}+3\delta T/2\\ 0\;T\geq T_{fu}+3\delta T/2 \end{array}\right.$$

In order to control the time steps, a free step backward differentiation formula with an automatic time step range of 1–2 was used [48]. The Newton method employing PARallel DIrect SOlver (PARDISO) solver was adopted [49,50,51]. A residual error of ~10^−6^ and a Newtonian damping factor of 0.8 were considered to solve the residual equations iteratively.

### 3.2. Mesh Sensitivity Analysis

**Figure 2:**
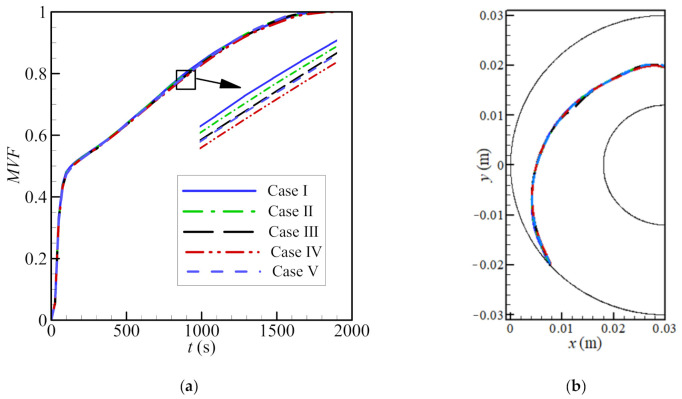
The impact of the mesh size on the melted volume fraction (MVF) of the phase change material (**a**) and the melting interface at a simulate time, *t* = 500 s (**b**) with a foam porosity *ε* of 0.80 (i.e., metal in the foam occupies 20% of the available volume), an eccentricity *e* of 0.22*r*_s_, and no nanoparticles (*σ* = 0.00).

**Figure 3:**
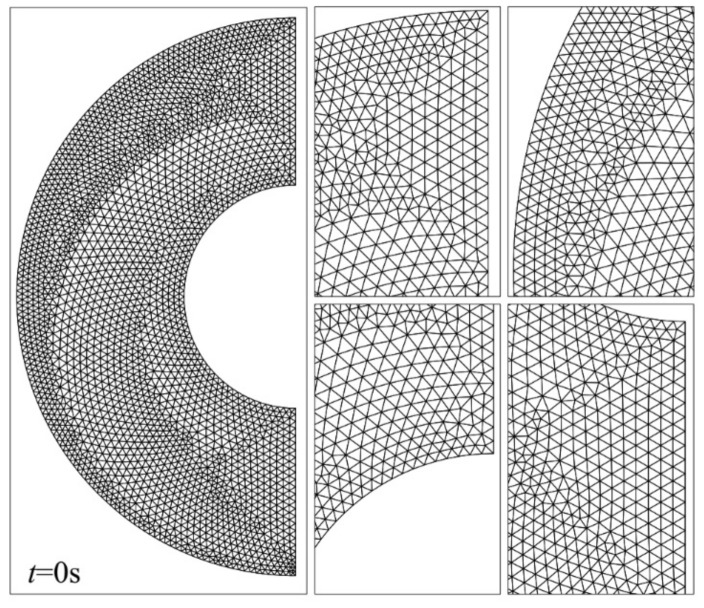
The grid of case III selected for the computations of the current analysis at *t* = 0 s.

**Figure 4:**
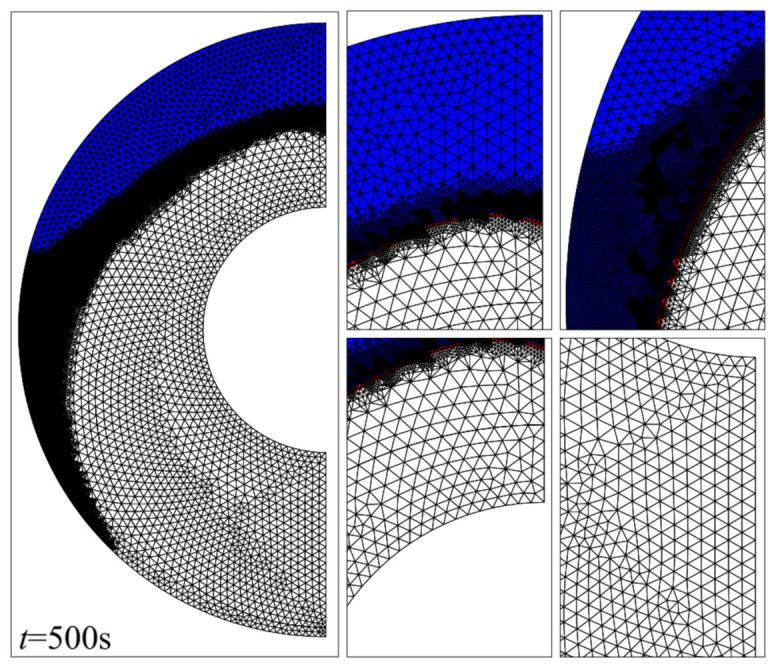
Mesh adaptation at *t* = 500 s. The white regions represent regions where the phase change material has melted, and the blue regions correspond to the solid zone. The apparent black region is a result of the mesh being too fine to resolve at this scale, such that only the element boundaries are visible.

Five different meshes were considered for testing the mesh sensitivity according to Table 2. As seen in Figure 2, the MVF and the melting interface after 500 s for the PCM free of nanoparticles (*ε* = 0.80, *e* = 0.22*r_s_*, and *σ* = 0.00) were used. There is a good agreement in all cases, especially cases III, IV, and V. Case III is chosen for further studies, as it is the least computationally expensive. There are 5104 elements in the domain, of which 335 lie at the boundaries. The mesh deformation and the adaptive refinement at *t* = 0 and *t* = 500 are demonstrated in Figure 3 and Figure 4, respectively. A desirable transient can be seen. Moreover, the results of the mesh sensitivity analysis showed a negligible error by using case III. Thus, the numerically uncertainty is negligible and will be not shown in the reported results.

### 3.3. Validation with the Literature

**Figure 5:**
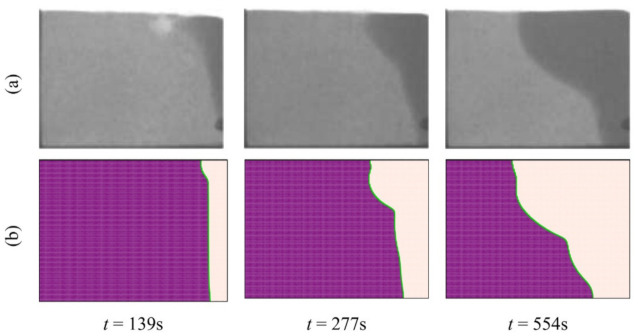
(**a**) The experimental observations of [52]; and (**b**) the numerical outcomes of the present investigation.

**Figure 6:**
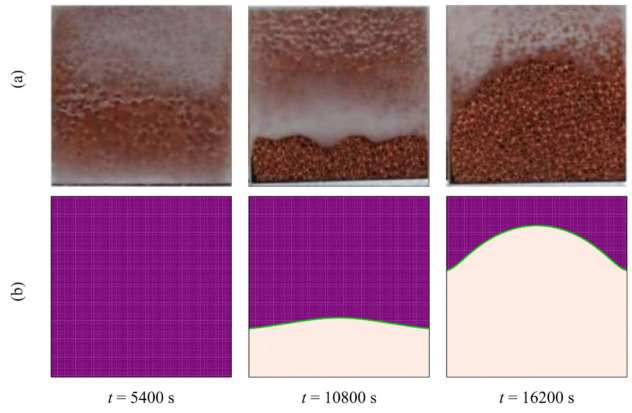
(**a**) The experimental observations in [45]; and (**b**) the numerical outcomes of the present study.

**Figure 7:**
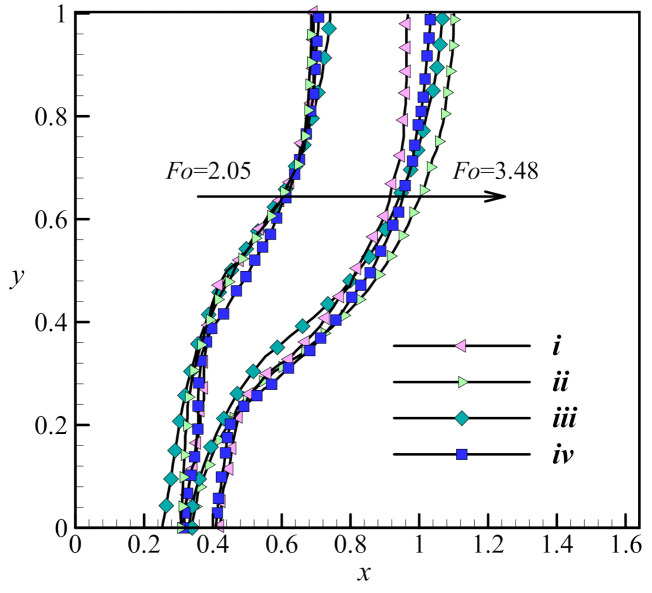
Comparison of the present computated melting lines to the review of [53]. i: the results of Kashani et al. [55], ii: the results of Gau and Viskanta (experiment); iii: the results of Brent et al.; and iv: the present study with *Fo* = 3.48 and 2.05, when the Prandtl number *Pr* = 50 and the Rayleigh number *Ra* = 1.25 × 10^5^.

**Figure 8:**
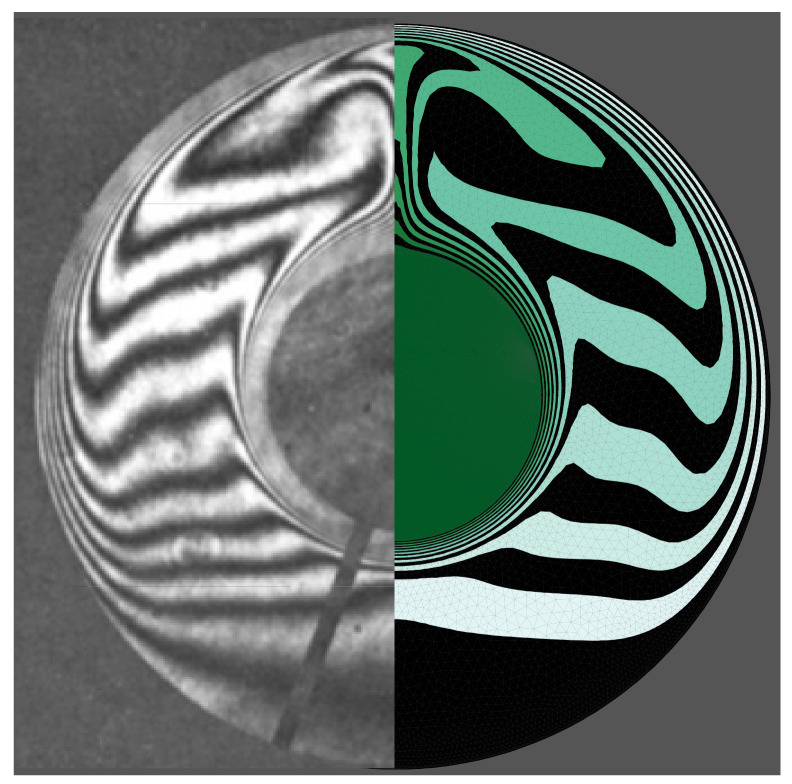
The isothermal contours of the present study (**left**) and [54] (**right**).

Previous experimental and numerical investigations have been used to validate the results and check the correctness of the numerical simulation of the present study. An experimental neutron flux study of the melting process and the evolution of the interface between the solid and liquid phases under a constant heat flux on the vertical walls of a square enclosure and other insulated walls gave an interface that has the same shape as is expected from the output of the simulation proposed in this study (Figure 5; [52]). A second study into the phase change of a paraffin wax in a copper foam with a porosity of 0.95 was compared to the output of the proposed simulation at three different time steps, and the shape of the interfaces was found to be similar in all three cases (Figure 6; [45]). A third study was also used, in which a pure (free of nanoparticles) PCM was melted in a square enclosure with the two side walls each held at a separate, constant temperature to establish a constant thermal gradient across the material. The results of that study were in agreement with the results of the model proposed here (Figure 7; [53]) at two non-dimensional times of *Fo* = 2.05 and 3.48. Finally, the calculated heat transfer by natural convection was compared to experimental results of the zone between two horizontal cylinders. The isotherm contours of two studies were compared at *Pr* = 6.19 and *Ra* = 2.33 × 10^5^ and show reasonable agreement (Figure 8; [54]).

## 4. Results and Discussion

The model was used to investigate the effects of eccentricity *e* within the range of 0–0.22 *r_s_*, porosity *ε* within the range of 0.8–1.0 (i.e., the metal foam occupies a volume between 20% and 0% of the available space, such that a porosity of 1 implies that no foam is present), the type of nanoparticles added, and the volume fraction *σ* of each type of nanoparticles ranging from 0.00 to 0.08.

**Figure 9:**
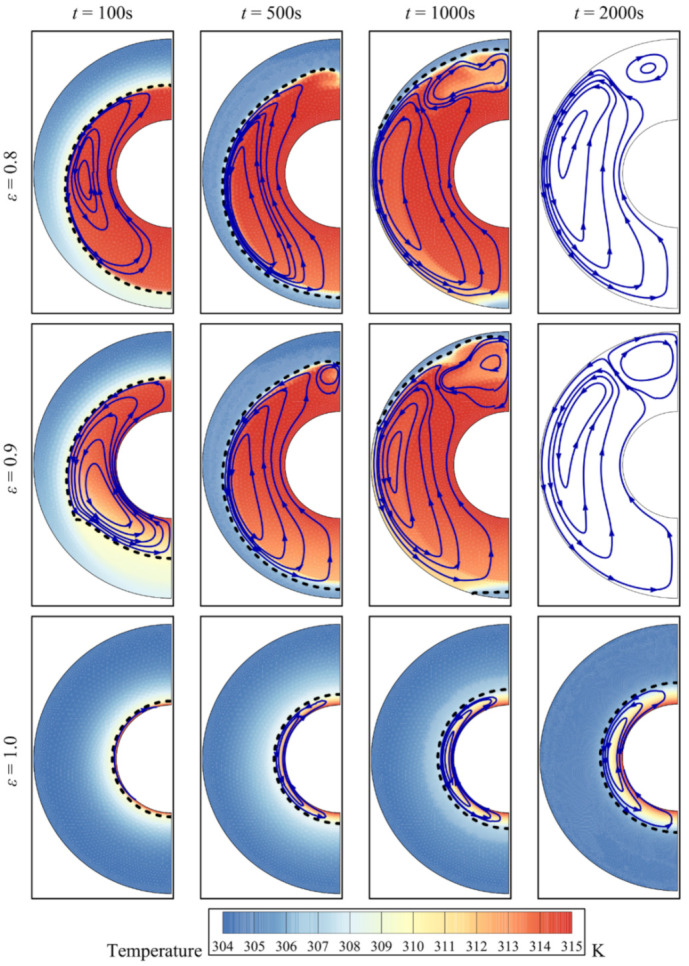
Isotherms, streamline, and melt interface for different porosity values at four charging times for Cu additives with an eccentricity *e* of 0.12*r*_s_ and a volume fraction of nanoparticles *σ* of 0.04.

When there is no foam (*ε* = 1), a layer of the melted PCM remains around the inner hot cylinder, while for the other values of *ε*, the PCM melts near the hot wall at first but the molten region then expands and covers the whole annulus (Figure 9). This implies that the presence of the copper foam contributes significantly to the melting of the PCM. Heat is transmitted by conduction within the foam, and this causes the PCM to melt. Once the PCM melts, convection in the melt is also able to transfer heat: hot liquid rises while cool liquid falls. Recirculation zones appear in the melt and surround the inner cylinder, which contributes further to melting.

**Figure 10:**
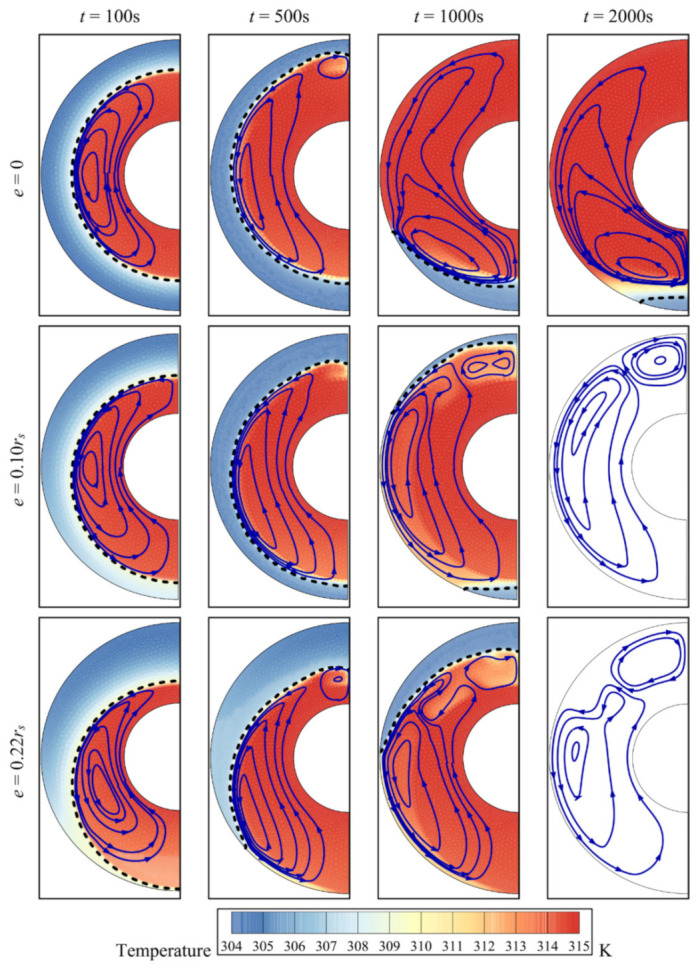
Isotherms, streamline, and interface of melting for different eccentricity values at four charging times for copper nanoparticles as additives with an eccentricity *e* of 0.12*r*_s_, a foam porosity *ε* of 0.8, and a nanoparticle volume fraction *σ* of 0.04.

In the case of a concentric annulus (no eccentricity, *e* = 0), the recirculation zone starts symmetric around the inner cylinder (Figure 10). As time progresses, convective effects become more pronounced and more portions of the PCM melt above the inner pipe and some remain solid below the inner tube. When eccentricity is increased, the porous zone around the inner pipe is shifted downwards. As the presence of the foam promotes melting as indicated earlier, all the PCM portions below the inner tube eventually melt. In the upper part of the cavity, the dominant convective effects also lead to full melting of the PCM in that region.

**Figure 11:**
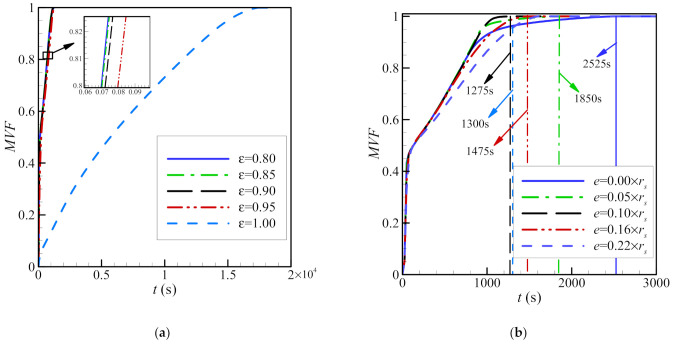
The effects of porosity (with an eccentricity *e* of 0.12*r*_s_ and a nanoparticle volume fraction *σ* of 0.04) (**a**) and eccentricity (with a porosity *ε* of 0.8 and a nanoparticle volume fraction *σ* of 0.04) (**b**) on the MVF for copper nanoparticles as additives.

**Figure 12:**
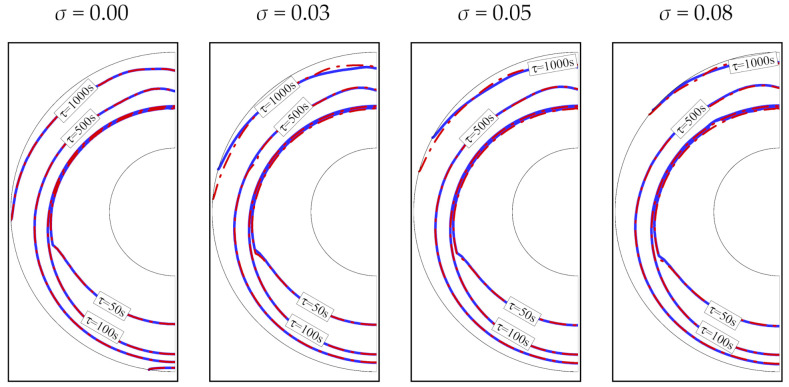
The interfaces of melting for different volume fractions of nanoadditives at four charging times with an eccentricity *e* of 0.12*r*_s_ and a porosity *ε* of 0.8. Solid lines are for graphite oxide nanoparticles, and dashed lines are for copper nanoparticles.

For a foam-free system (*ε* = 1), the melt volume fraction increases slowly with time. For the other values of *ε*, the melt volume fraction increases much faster. This is because a lower value of *ε* implies a greater presence of the solid foam and, consequently, more heat transmission throughout the PCM, which ultimately leads to melting (Figure 11). It is also shown that for a concentric system (*e* = 0), the time required to achieve complete melting is higher, compared to that in an eccentric system. This is related to the observations shown in Figure 10. When there is no eccentricity, convection enhances melting in the upper region where hot liquid is ascending, while at the bottom of the annulus, melting is slow as the copper foam does not fully cover that region. When eccentricity is greater, the PCM melts faster in the bottom region, while melting also continues in the upper region due to free convection (Figure 12). The full melting time is approximately halved in a system with an eccentricity equivalent to 0.1*r*_s_, compared to in a concentric system.

**Figure 13:**
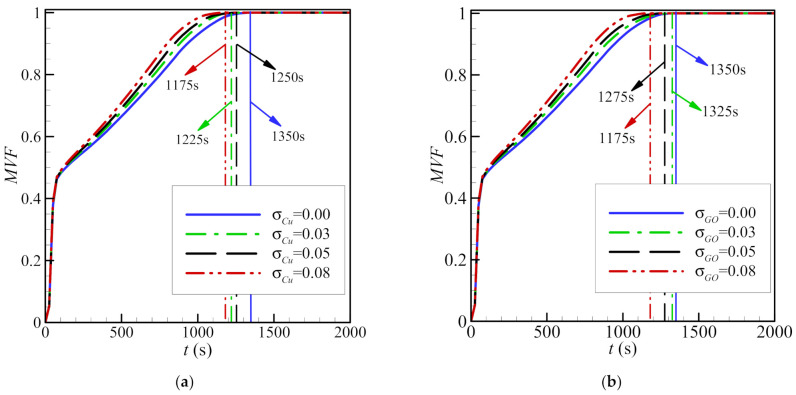
The effects of various volume fractions of copper nanoparticles (eccentricity *e* = 0.12*r*_s_ and porosity *ε* = 0.8) (**a**) and various volume fractions of graphite oxide nanoparticles (eccentricity *e* = 0.12*r*_s_ and porosity *ε* = 0.8) (**b**) on the MVF.

For a greater fraction of nanoparticles (increased *σ*), the melting front is more developed and closer to the outer cylinder, indicating an increase in the molten PCM. This is true for both copper and graphite oxide nanoparticles. The nanoparticles dispersed in the PCM improve heat transfer. The type of nanoparticles has a limited impact on the melting front, as the location of the front remains almost the same for both graphite oxide and copper nanoparticles, as does the volume fraction of the PCM (Figure 13). As seen, using 8% of nanoparticles could reduce the melting time by 13%, caculcated by 100 × (1350−1175)/1350 for both types of nanoparticles.

Initially, the volume fraction of the molten PCM increases sharply, as the conduction in the copper foam is dominant at short times. The volume fraction of the molten material then increases gradually when convection dominates, until melting is complete. In all cases, the time to complete melting is lower, when nanoparticles are present. The melting time is minimized, when the amount of nanoparticles is greatest (*σ* = 8%). Total melting occurs faster when copper nanoparticles are used compared to that when graphite oxide particles are used.

**Figure 14:**
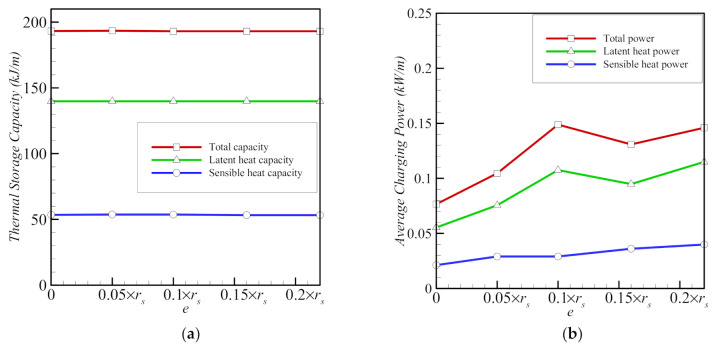
Variations of average thermal storage capacity (**a**) and average charging power (**b**) as functions of eccentricity *e* for copper nanoparticles for porosity *ε* = 0.8 and nanoparticle volume fraction *σ* = 0.04.

The latent storage capacity and charging power are greater than the sensible capacity and power (Figure 14), as most of the involvement of the PCM in the heat transfer is consumed by the melting process. It is shown that the latent and sensible storage heat capacities are almost the same for all the values of eccentricity since the phase change mass is constant. Since the stored heat is constant, the charging power is only sensitive to the melting rate. Melting is accelerated due to the presence of the porous foam around the inner pipe and convection in the upper region of the cavity, and *e* = 0.1*r*_s_ corresponds to the optimal placement of tube under those conditions, compared to zero eccentricity. Conversely, when the inner pipe and the surrounding porous zone are concentric, the melting time is greatest, and consequently, the average charging power is lowest.

**Figure 15:**
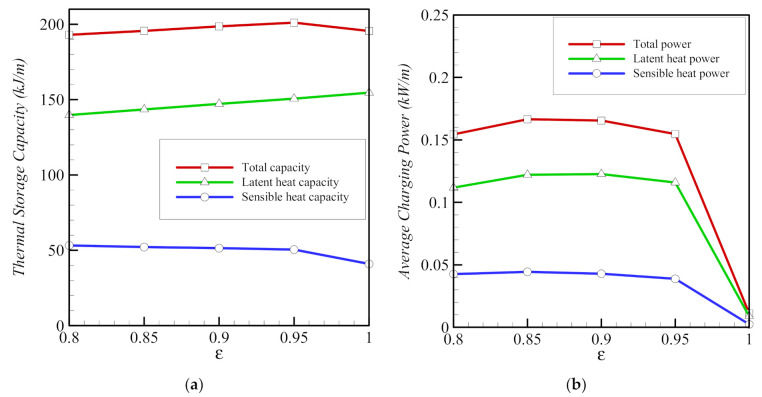
Variations of average thermal storage capacity (**a**) and average charging power (**b**) as functions of porosity *ε* for copper nanoparticles for eccentricity *e* = 0.12*r*_s_ and nanoparticle volume fraction *σ* = 0.04.

Reducing porosity (raising *ε*) leads to a slight reduction in the sensible storage capacity and an increase in the latent capacity (Figure 15). The average charging power decreases sharply to almost zero, when there is no foam (*ε* = 1). In this case, the total melting time is very long, as no heat is transmitted by the metal foam, which is consistent with the data in Figure 9 and Figure 11.

**Figure 16:**
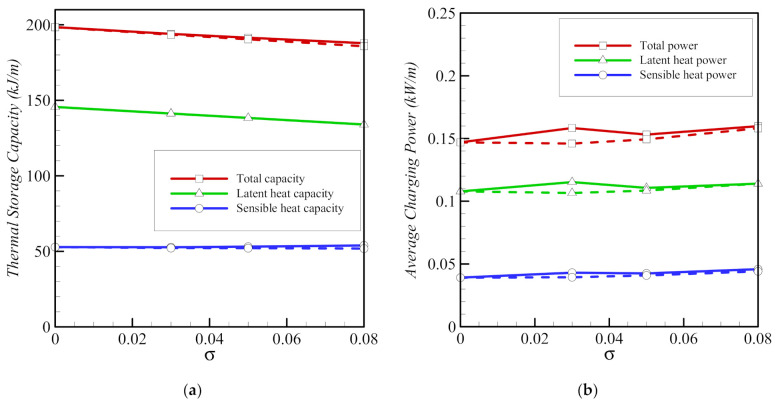
Variations of average thermal storage capacity (**a**) and average charging power (**b**) as functions of the volumetric fraction of copper nanoparticles indicated by solid lines and graphite oxide nanoadditives indicated by dash lines for porosity *ε* = 0.80 and eccentricity *e* = 0.12*r*_s_.

A moderate decrease in the storage capacity is observed, when the nanoparticle content *σ* is increased for both copper and graphite oxide (Figure 16). Charging power is higher when copper nanoparticles are used, compared to when graphite oxide nanoparticles are used. This is consistent with the observation that the total melting time is lower in the case of copper nanoparticles and when a lower volume fraction of nanoparticles is used (Figure 13).

## 5. Conclusions

A shell-and-tube LHTES system was modeled, and its performance was simulated. The inner tube was supported by a layer of copper metal foam. Graphite oxide and copper nanoparticles were tested as additives to accelerate heat transfer in a PCM. The impact of convective heat transfer in the molten region was taken into account. The outcomes of the present numerical analysis can be summed up as follows:

- The presence of a metal foam around the inner pipe contributes significantly to heat transfer and to the melting of the PCM. Reducing the porosity of the foam increases the amount of heat transmitted through the foam and enhances the melting of the PCM.
- The eccentricity of the system affects heat transfer by changing the location of the foam around the inner pipe. The melting of the PCM is limited by convection in the upper part of the cavity and by conduction through the metal foam in the lower region of the cavity. The melting of the PCM is slowest, when the inner pipe and the porous zone are concentric; the total melting time is halved for the eccentricity *e* = 0.1*r*_s_.
- Using a higher volume fraction, *σ*, of nanoparticles enhances melting. Copper nanoparticles are slightly more effective in transferring heat than graphite oxide nanoparticles. For both types of nanoparticles, the time required to melt the PCM completely is 13% shorter for an 8% volume fraction of nanoparticles (*σ* = 0.08).
- The three parameters, the porosity ε, the eccentricity *e*, and the volume fraction of nanoparticles, *σ*, have limited effects on the total storage capacity. However, the average charging power and the total melting time are affected more strongly by these parameters. Overall, the charging power decreases for longer total melting time, mainly for smaller values of eccentricity *e* and for lower volume fractions of foam (higher values of *ε*). The charging power is highest, when eccentricity takes the value *e* of 0.1*r*_s_.

## Figures and Tables

**Table 1:** Thermophysical characteristics of the materials used in this study [37,38].

Material	*P* (kg·m^−3^)	*C*_p_ (J·kg^−1^·K^−1^)	*β* (K^−1^)	*T*_fu_ (°C)	*λ* (W·m^−1^·K^−1^)	*h*_sf_ (kJ·kg^−1^)	*μ* (m^2^·s^−1^)
**PCM**	Solid: 1018 Liquid: 888	Solid: 1900 Liquid: 2400	1.51 × 10^−3^	32	Solid: 0.372 Liquid: 0.153	152.7	3 × 10^−6^
**Metal foam**	8900	386	/	/	380	/	/
**Graphite oxide**	1800	0.717	2.84 × 10^−4^	/	5000	/	/
**Copper**	8933	0.385	1.67 × 10^−5^	/	401	/	/

**Table 2:** Mesh details for cases in the mesh sensitivity study. Case III was used for subsequent calculations.

Cases	Elements in the Domain (Boundary)	Time to Run
**Case I**	2246 (276)	20 min 30 s
**Case II**	3703 (312)	32 min 4 s
**Case III**	5104 (335)	43 min 52 s
**Case IV**	20,153 (801)	1 h 48 min 47 s
**Case V**	50,092 (1895)	3 h 34 min 9 s

## Data Availability

Data is contained within the article.

## Nomenclature

*A*	surface area of domain, m^2^
*A*_mush_	mushy zone parameter, Pa·s·m^−^^2^
*C_p_*	specific heat capacity, J·kg^−1^·K^−1^
*d_l_*	ligament diameter of foam, m
*d_p_*	pore diameter of foam, m
*e*	eccentricity, m
*ES*	total energy stored, k·m^−1^
*g*	gravitational acceleration, m·s^−2^
*h_sf_*	interfacial heat transfer coefficient, W·m^−2^·K^−1^
*MVF*	melting volume fraction
*n*	normal direction of the surfaces
*p*	pressure, Pa
*Pr*	Prandtl number
*R*	radial coordinates, m
*Ra*	Rayleigh number
*r_h_*	radius of the heated tube, mm
*r_p_*	the radius of the average porous layer, mm
*r_s_*	radius of the shell of LHTES unit, mm
*s*	source term
*T*	temperature field, K
*t*	time, s
*T*_c_	initial temperature of LHTES unit, K
*T*_h_	temperature of hot wall, K
*u, v*	*x*- and *y* -direction velocities, respectively, m·s^−1^
*Fo*	nondimensional time (*tρC*_p_)/(4*λr*_s_^2^)
**Greek**	
*µ*	dynamic viscosity, kg·s^−1^·m^−1^
*β*	coefficient of thermal expansion, K^−1^
*δT*	temperature increment window for fusion, K
*Δλ*	thermal conductivity difference, W·m^−1^·K^−1^
*ε*	porosity
*η*	liquid volume fraction
*η*_0_	dummy liquid volume fraction for an adaptive mesh
*ϑ*	computational constant
*κ*	permeability, m^2^
*λ*	thermal conductivity, W·m^−1^·K^−1^
*ρ*	density, kg·m^−3^
*σ*	volume fraction of nanoadditives
*τ*	nondimensional time
*χ*	a variable defined as (1 − *ε*)/(3π)
*ω*	pore density (PPI)
**Subscripts**	
cz	clear zone (no metal foam)
eff	effective
fu	fusion
*i*	index variable
*j*	index variable
l	liquid
m	metal foam
mush	mushy zone
NePCM	nano-enhanced phase change material
PCM	phase change material with no nanoadditive
pz	porous zone (metal foam)
s	solid
